# Genome plasticity driven by aneuploidy and loss of heterozygosity in *Trypanosoma cruzi*

**DOI:** 10.1099/mgen.0.000843

**Published:** 2022-06-24

**Authors:** Lissa Cruz-Saavedra, Philipp Schwabl, Gustavo A. Vallejo, Julio C. Carranza, Marina Muñoz, Luz Helena Patino, Alberto Paniz-Mondolfi, Martin S. Llewellyn, Juan David Ramírez

**Affiliations:** ^1^​ Centro de Investigaciones en Microbiología y Biotecnología-UR (CIMBIUR), Facultad de Ciencias Naturales, Universidad del Rosario, Bogotá, Colombia; ^2^​ Institute of Biodiversity, Animal Health & Comparative Medicine, University of Glasgow, Glasgow G12 8QQ, UK; ^3^​ Laboratorio de Investigación en Parasitología Tropical, Facultad de Ciencias, Universidad del Tolima, Ibagué, Colombia; ^4^​ Molecular Microbiology Laboratory, Department of Pathology, Molecular and Cell-Based Medicine, Icahn School of Medicine at Mount Sinai, New York, NY 10029, USA

**Keywords:** *Trypanosoma cruzi*, TcI, genome, aneuploidy, segmental allele frequency, loss of heterozygosity

## Abstract

*Trypanosoma cruzi* the causative agent of Chagas disease shows a marked genetic diversity and divided into at least six Discrete Typing Units (DTUs). High intra genetic variability has been observed in the TcI DTU, the most widely distributed DTU, where patterns of genomic diversity can provide information on ecological and evolutionary processes driving parasite population structure and genome organization. Chromosomal aneuploidies and rearrangements across multigene families represent an evidence of *T. cruzi* genome plasticity. We explored genomic diversity among 18 Colombian *T. cruzi* I clones and 15 *T*. *cruzi* I South American strains. Our results confirm high genomic variability, heterozygosity and presence of a clade compatible with the TcI_dom_ genotype, described for strains from humans in Colombia and Venezuela. TcI showed high structural plasticity across the geographical region studied. Differential events of whole and segmental aneuploidy (SA) along chromosomes even between clones from the same strain were found and corroborated by the depth and allelic frequency. We detected loss of heterozygosity (LOH) events in different chromosomes, however, the size and location of segments under LOH varied between clones. Genes adjacent to breakpoints were evaluated, and retrotransposon hot spot genes flanked the beginning of segmental aneuploidies. Our results suggest that *T. cruzi* genomes, like those of *Leishmania*, may have a highly unstable structure and there is now an urgent need to design experiments to explore any potential adaptive role for the plasticity observed.

## Data Summary

Data generated in this work were deposited on ENA under the bioproject PRJEB48841. All supporting data were included in supplementary data files, three supplementary figures and three tables are available with the online version of this article.

**Impact Statement**

The high genetic variability of *T. cruzi*’s TcI strains and its possible relationship with recombination events has been one of the great challenges in the study of this parasite. Recent genomic analyses on this parasite demonstrated meiotic sex across its genome and multigene family’s re-arrangement associated with recombination processes. We found genome evidence of high variability and plasticity in TcI clones, where events of chromosomal and segmental aneuploidy and loss of heterozygosity play an important role. Our findings are important to support TcI high genomic variability, genomic plasticity and open new questions to understand the drivers of genomic structural instability present in this neglected parasite.

## Introduction

*Trypanosoma cruzi* is a flagellated protozoan that causes Chagas disease, which affects approximately 6 to 7 million people worldwide [1]. This parasite exhibits high genetic variability and is classified into six Discrete Typing Units (DTUs), being *T. cruzi I* (TcI) the most widely distributed DTU [2–4]. The high genetic variability of *T. cruzi* I has been previously described using several approaches. In turn, the presence of a unique clonal genotype emerges associated with human infection in Colombia and Venezuela, known as TcI_dom_ [5–11].

For several years, the *T. cruzi* genome has been thought to be highly plastic, even before the arrival of genome sequencing. Dvorak and colleagues clearly demonstrated substantial inter-clonal variation in DNA content as early as the 1980s employing cytometry strategies that have been corroborated by recent studies [12, 13]. Analyses of *T. cruzi* genomes with micro-array technologies drew similar conclusions, with reports of substantial copy number variation and aneuploidy among closely related *T. cruzi* clones [14]. Subsequent genomic studies, many based on a limited number of laboratory strains, have divided into a disruptive compartment and a core compartment, and confirmed high levels of genome plasticity in *T. cruzi* mainly associated with the disruptive compartment [15–17]. Hi-C sequencing suggest that chromosomal locations and distributions of gene families, especially those that are highly repetitive and encode surface-expressed products, are difficult to predict and highly fluid in nature [15].

Genomic instability, principally aneuploidy, has been described in multiple microorganisms [18–22], as well as being widely reported in cancer [23]. In some unicellular fungi and protozoa, genomic plasticity occurs as means of adaptation to environmental stress [19–21]. Common manifestations of genomic plasticity include whole and segmental aneuploidy, as well as copy number variation in some genes. Trypanosomatids exercise little control over transcription [24]. As such, copy number variation can have profound consequences on gene expression, with important adaptive implications [25]. The alternate mitotic and spindle assembly checkpoints (MACs and SACs, respectively) in trypanosomatids are considered basal to higher eukaryotes and could contribute to the high rate of aneuploidy observed in some species [26]. Ploidy instability is particularly well-described in *Leishmania* sp., linked to drug resistance and metabolic plasticity [27, 28]. Intriguingly, aneuploidy is infrequent or absent in *T. b. brucei* [29]. In *T. cruzi* little is known about the frequency and adaptive significance of aneuploidy, although naturally occurring aneuploids are increasingly reported [14, 15, 30, 31].

*Trypanosoma cruzi* has recently shown to undergo frequent recombination [5, 30, 32]. Reports of preponderant clonal evolution in this parasite where the recombination events are not frequent between the populations to break clonal patterns, are a gross over-simplification based on antiquated genetic approaches [33, 34]. In fact, *T. cruzi* DTU I, at least, appears to have a complex metapopulation structure with a mosaic of mating systems simultaneously present – clonal, sexual and parasexual [30]. Recombination not only provides a source of rapid phenotypic innovation, but also can have fundamental impacts on genome structure – especially where parasexual and/or non-canonical meiosis are at play (e.g. [35]). Aneuploidy observed in *Trypanosoma cruzi* could arise from just such processes (e.g. [14, 30, 31]). Experimental *T. cruzi* DTU I hybrids created, in the laboratory are thought to have arisen via parasexual genome fusion resulting in sub-tetraploid progeny [36]. A genomic fusion-then-loss mechanism similar to that observed in yeasts [37] has been proposed to explain patterns of aneuploidy observed in *T. cruzi* using experimental hybrids [36].

To explore the diversity and drivers of genome plasticity in *T. cruzi*, we sequenced the genomes and explore the resultant karyotypes of 18 *T*. *cruzi* DTU I genomes from Colombia. Colombian clones were assessed alongside reference genomes from around South America. We found differences in TcI strains whole-genome SNPs (single nucleotide polymorphism) number and heterozygosity that are not necessarily related to geographical and host isolation. Important events of aneuploidy around the whole and segmental parts of chromosomes, and events of LOH, were not necessarily represented in all the clones per strains. The results here described shows a tremendous *T. cruzi* genomic plasticity.

## Methods

### Maintenance of parasites

A total of five *Trypanosoma cruzi* strains isolated from different parts of Colombia maintained during approximately 10 to 20 passages since their isolation, and previously characterized as TcI, were cultivated in liver infusion tryptose (LIT) medium supplemented with 10 % fetal bovine serum and incubated at 26 °C until the start of the experiments. The origin of the strains is found in Table S1 (available in the online version of this article).

### Cell cloning - cell sorting

Multiclonality has already been described in *T. cruzi* strains [30, 38]. Log phase epimastigote cultures of *T. cruzi* were washed in 1X PBS and maintained until the start of the flow cytometry protocol for cell sorting cloning. The parasites were drawn on the BD FACS Aria II equipment, using the BD FACSDiva software (Becton, Dickinson Biosciences). In summary, 1 ml of epimastigotes in 1X PBS was drawn directly into 96-well plates at a concentration of one parasite per well and 50 µl of LIT medium supplemented with 10 % fetal bovine serum and penicillin/streptomycin was immediately added to 2 %. We checked for the viability every day until we got a mass cultivation. A total of 40 clones were generated for each strain, the above in order to increase the number of clone recoveries. The viability of the parasites was verified under an inverted microscope, when an increase in the concentration of the parasites was observed, LIT medium was added in volumes of 50 µl, when the culture exceeded 200 µl, it was massified in a 25 cm^3^ culture box and maintained as previously described. Approximately five clones were selected for each strain. Each clone was genotyped using the algorithm proposed by Ramírez *et al.,* 2010 [39]. A total of 18 clones were finally selected for further analysis.

### DNA extraction and sequencing

DNA extraction was performed from epimastigote cultures in LIT medium in the logarithmic phase at a concentration of approximately 1×10^6^ parasites/ml after one passage since they were cloned and massified. The parasites were washed twice with 1X PBS. The DNeasy Blood and Tissue kit from Qiagen was used (catalogue No. 69504; Qiagen, Hilden, Germany). A volume of 200 µl of Buffer AL, 20 µl of proteinase K and 1 µl of RNAse A were added to the parasite pellet, the content was re-suspended and incubated at 36 °C for 20 min in order to degrade the proteins and the RNA not required. A second incubation was carried out at 56 °C for 10 min, at the end of this time, the provider’s protocol was followed. A total of 100 µl of DNA was obtained. The concentration and purity of DNA was verified by means of a measurement in NanoDrop 2000/2000 c Spectrophtomers (ThermoFisher scientific), a concentration greater than 1 mg ml^−1^ and a value of 2±2 for the 260/280 and 230/260 indices. They were considered successful, and the integrity of the DNA was evaluated by means of a 2 % agarose gel electrophoresis. The extracted DNA that met all the mentioned quality parameters was sent to Novogene Bioinformatics Technology Co., Ltd, Beijing, China, for sequencing using Illumina’s HiSeq X-Ten system platform, mate-paired libraries were built using end repair (350 bp insert size) and subjected to paired-end sequencing (2×150 bp read length). The reads obtained were filtered by adapter contamination, >10 % uncertain nucleotides, or >50 % low-quality nucleotides (base quality <5), approximate depth was 60X and 2.5 GB of data.

### DNA mapping and variant calling

The quality of the reads obtained for the 18 sequenced genomes was evaluated using the fastQC software (https://www.bioinformatics.babraham.ac.uk/projects/fastqc). The sequenced genomes were mapped using the BWA-mem v0.7.3 software (Burrows-Wheeler Aligner) under the default parameters, using the *T. cruzi* – Brazil A4v49 genome as reference [15, 40]. The non-mapping reads were mapped using the maxicircle genome Sylvio-x10, because of its availability, to corroborate the absence of those reads in our analysis. Subsequently, the Samtools sort v0.1.18 tool was used to sort the alignments, followed by the marking of the PCR-duplicates by Picard v1.85 [41]. SNPs analysis was performed with Genome Analysis Toolkit (GATK) v3.7.0 using the HaplotypeCaller option [42]. The output files obtained for each clone were linked using GATK GenotypeGVCFs. The obtained vcf file was filtered by depth (DP <10>500), quality of the reads (QUAL <1500), and finally, the selectType option of GATK was used to obtain the SNPs. Additionally, bedtools intersect was used to place a virtual mappability mask to exclude the variants present in unreliably mappable regions from the *T. cruzi* – Brazil A4v49 reference genome, following the protocol performed by Schwabl *et al.,* 2019 [30]. The reads per sample per chromosome calculation were made using samtools view options -f 1 F 12.

### Phylogenetic reconstruction

The vcf file that contains the information for the SNPs present in the sequenced genomes for all clones regarding the nuclear genome of Brazil A4 were used to create multifasta file using vcf-to-tab, excluding sites with missing information (--max-missing option). MAFFT v7.271 was used to perform the alignment [43]. The reconstruction of nuclear phylogeny was performed by maximum likelihood estimation in IQ-TREE v1.5.4, using an initial search for the best substitution model applied to the sequences and 1000 ultrafast bootstrap replicates [44]. The files were viewed in FigTree v1.4.3 and edited in the Interactive Tree of Life (iToL) tool [45]. Values of heterozygosity were determined using vcftools -het option that calculated the F index. The count of SNPs was executed with bcftools view options -c1 -H. A total of 15 nuclear sequences corresponding to TcI reference genomes isolated from different geographical points of America were included (Table S1).

### Determination of chromosomal somy

For the determination of somy, the standardized protocol by Schwabl *et al*., 2019 was used [30]. Briefly, the Samtools depth tool v0.1.18 was used to determine the average depth for 1 kb windows along each of the chromosomes from the .bam files obtained from the mapping by BWA, followed by this the median of the averages was calculated for the previously obtained windows [46]. Finally, the estimation of the somy was made divided by the calculated median n the 40th percentile and multiplying by two [30]. The results obtained were graphed using the heatmap.2 function from gplots package in the R v3.6.3 software.

### Allelic frequency and loss of heterozygosity (LOH)

The data corresponding to the allelic frequency (AF) were purified in text files from the SNPs file using vcftools and SelectVariants - VariantsToTable of GATK for the genome of each one of the clones, and plotted using the plot function of R [42], in disomic patterns the AF is reflected in a heterozygosity radius of 0 or one for homozygous SNPs and 0.5 for heterozygous SNPs, when there is a trisomic pattern a heterozygosity radius of close to 0.66 and 0.33 is observed, and in tetrasomic patterns the radius of heterozygosity is close to 0.25 and 0.75. For LOH determination, the vcf file containing the information for SNPs was separated into SNPs, positions and individuals using vcftools. The above files were used as input files in R v3.6.3 software, where data, table, stringr, ape, and phangorn packages were used. Heterozygous, homozygous distinct to the reference, and homozygous SNPs equal to the reference genome were determined for each position. SNPs present in hard-to-map regions of the reference genome were excluded from the analysis. The number of SNPs per chromosome were plotted using 10 kb windows using R plot.

### Identification of genes across the segments

The IDs for the genes present in regions that exhibited changes in the allelic frequency - relevant depth and areas with LOH, were extracted from the gff file for the Brazil A4 v49 reference genome and subjected to the TriTrypDB tool. using an initial search in the Brazil A4 genome followed by a gene orthology analysis, in order to cover data on genes noted as coding for nonspecific proteins [47]. The list of genes present in these areas was obtained and purified by means of dynamic tables in Excel.

## Results

### Phylogenetic clustering and multiclonality

**Fig. 1.**
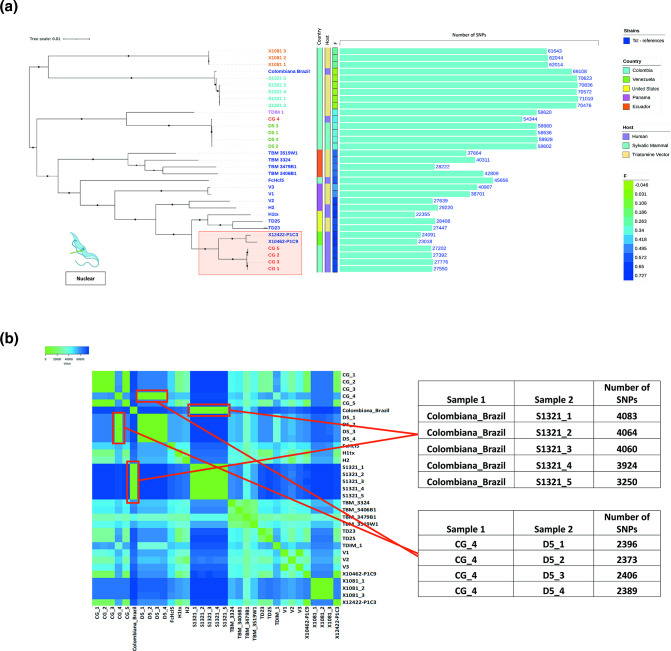
Phylogenetic reconstruction of *Trypanosoma cruzi* I. (a) Phylogenetic reconstruction from *Trypanosoma cruzi* I genomes isolated from the Americas. Blue colour: TcI reference clones, different colours: Colombian TcI clones . Country origin is represented with different colours, homozygosity values in the heatmap and SNPs number in the barplot. The corresponding cluster with the TcI_dom_ genotype is indicated in the phylogeny by red square. (**b) **SNPs differences per clones and strains. The heatmap shows the the tables contain the number of SNPs differences between Colombiana-Brazil and 1321 clones, and CG_4 clone and D5 clones.

A total of 468521 SNPs relative to the *T. cruzi* BrazilA4 reference genome sites were identified across all strains and clones which were included relative to the *T. cruzi* BrazilA4 reference genome. *T. cruzi* Brazil A4 reference genome was employed because of the low number of gaps, good annotation, and assembly per chromosomes in comparison with other TcI reference genomes reported until the moment. To evaluate taxonomic affinities within *T. cruzi* I, a phylogenetic tree was constructed using the maximum likelihood method. We included 15 *T*. *cruzi* clones isolated from different geographical locations of the Americas (blue lines) and 18 TcI clones generated from Colombian strains (colour per strain) (Table S1). We observed two clusters, one of them related to the clones of 1321, X1081, and Colombiana-Brazil, and the other that included the rest of the clones. Additionally, tree topology correlated with geography, with most Colombian clones forming a discrete cluster separate from strains of other countries. However, some exceptions were found in this analysis (Fig. 1). The first of them corresponded with the Colombian cluster that included the strain Colombiana-Brazil and S1321 and X1081 clones, showing the highest number of SNPs compared to the reference and the lowest inbreeding coefficient (F). Interestingly Colombiana-Brazil and S1321 cluster together with between 3000–4000 different SNPs approximately, with the clone S1321-5 having the fewest differences (3250 SNPs) (Table S2, Fig. 1b). Four clones of the CG strain [1–3, 5] clustered alongside TcI_dom_ clones X10462-P1C9 and X12422-P1C3, isolated from Venezuela (with robust bootstrap support (100%) (red square)), with the highest F index and low number of SNPs (Fig. 1a). Bootstrap values higher than 80 are represented with a black circle in the tree.

Divergent clones within single hosts were observed, especially from a human sample (CG) where four clones were associated with TcI_dom,_ as we previously described. While CG-4 was more closely linked with parasites of sylvatic origin as D5, which is in correspondence with the cluster that shows a different number of SNPs and F index in comparison with the TcI_dom_ cluster, when we calculated the amount of different SNPs between CG_4 and the other CG clones it was approximately 58000 SNPs (Fig. 1b, Table S2). No clear association between the origin of the isolation (human, mammalian host and vector) and the phylogenetic groups was found across the full dataset, although the clade including 1321, X1081 and Colombiana-Brazil strains represented only sylvatic hosts (Fig. 1a).

### Chromosomal aneuploidy in *T. cruzi* I clones

**Fig. 2.**
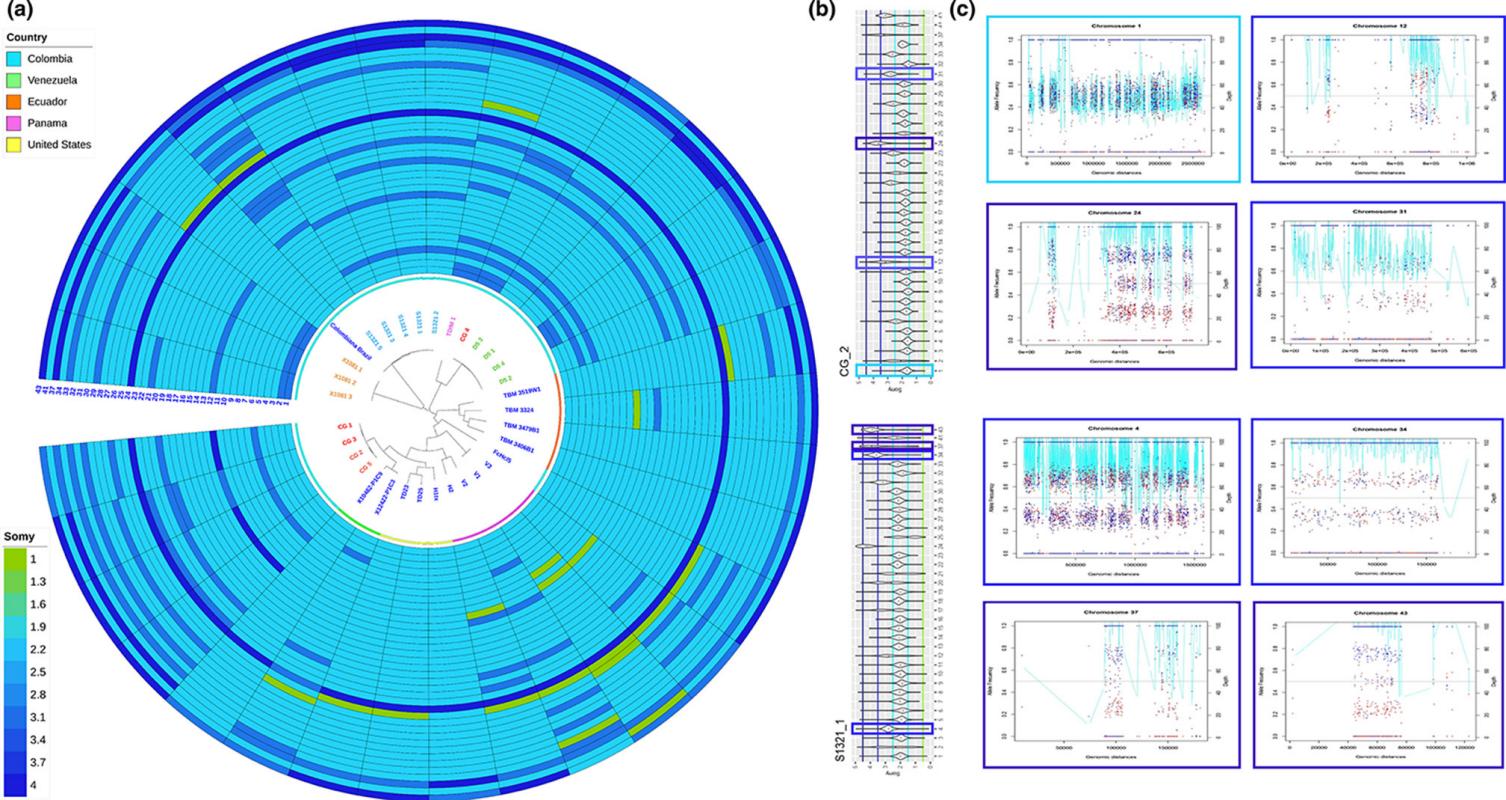
Chromosomal aneuploidy in *T. cruzi* I clones. (a) The heatmap shows the ploidy calculated from the depth per chromosome for each of the TcI clones. Reference clones and sequenced clones for this study are noted. The phylogenetic reconstruction in the middle, obtained from SNPs data, follows the same characteristic Fig. 1. (b) Violin graphs show depth mean per chromosome, green, light blue, blue and dark blue lines indicate monosomic, disomic, trisomic and tetrasomic chromosomes respectively. (**c) **Depth (light blue lines) and allelic frequencies (red and blue points) for the chromosomes.

Aneuploidy was considered when changes were found in the number of chromosomes involving the entire chromosome. Substantial somic heterogeneity (Fig. 2a, b (light blue lines inside the plot)), inferred from depth (light-blue lines), and validated using alternate allele frequency (AAF) calculation (red and blue points) (Fig. 2b), was observed in the TcI clones. Examples of alternate allele frequency plots (more than three chromosome copies) based on karyotype estimates are shown in Fig. 2C. Karyotype was not clearly correlated with phylogeny or geography (Figs 2a and S1). Instead, the strain origin of the clones was an apparent driver of karyotype among some Colombian clones. The most affected chromosomes concerning changes in somy during this analysis were chromosomes 24, 37 and 43 that showed tetraploid characteristics for most of the clones (Fig. 2a). Chromosome 24 was found to be pentasomic in D5 clones and CG-4. Chromosomes 35, 36, 38, 39, 40, 41 and 42 were excluded from the analysis given the percent of repeated content in the Brazil A4 reference genome and consequently, with a high masking. Finally, the clones TD23 and TD25 (external data) were the only ones that do not show a relationship between the depth (disomic) and AAF (trisomic), possibly related with depth during the sequencing (Fig. S2).

### Segmental aneuploidy in *Trypanosoma cruzi*

**Fig. 3.**
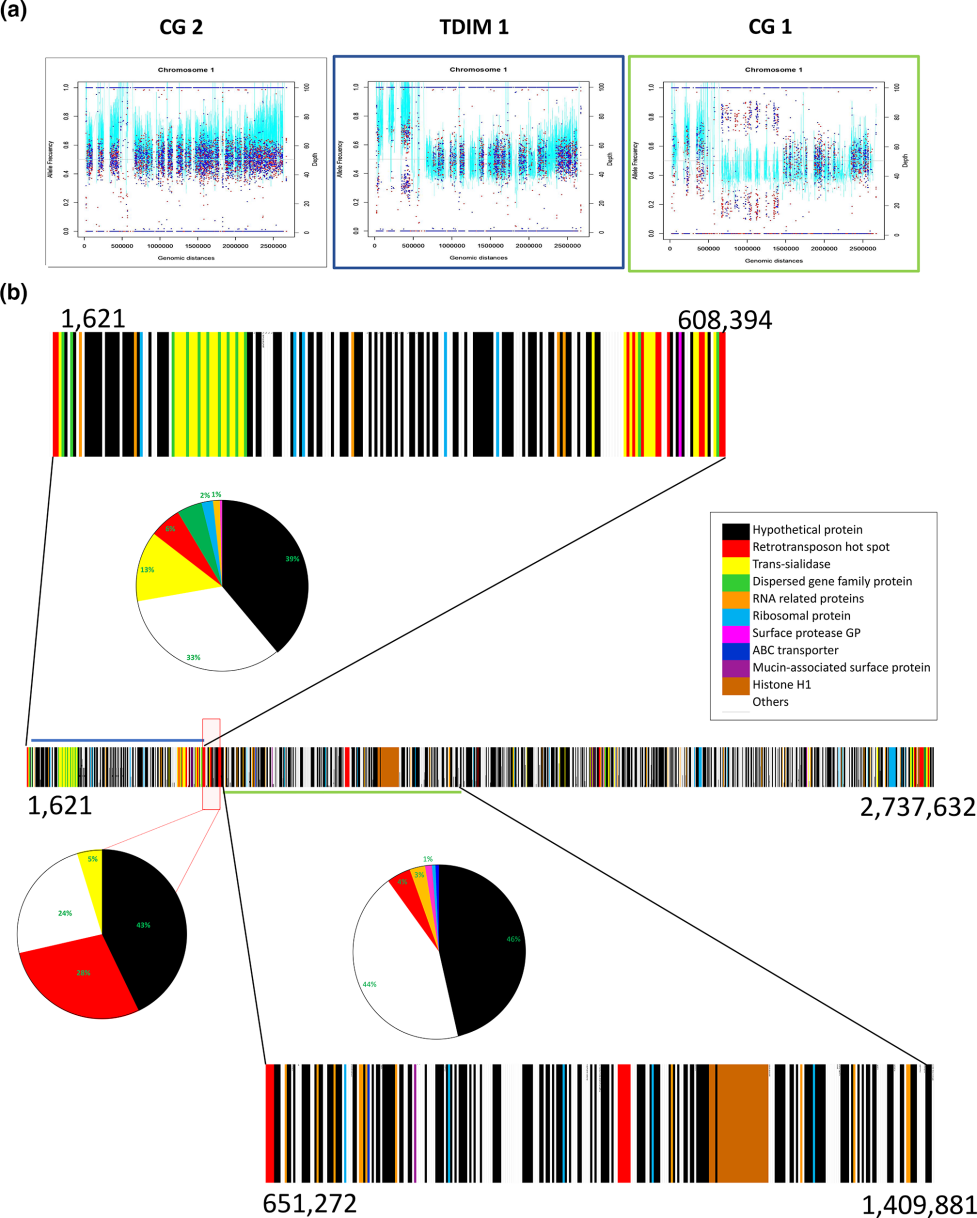
Segmental modifications in allelic frequency and depth of chromosome 1 and gene annotations. (a) The graph shows the patterns of segmental allelic frequency and depth observed in the TcI clones evaluated, depth (light blue lines) and allelic frequencies (red and blue points) for the chromosomes, the graph of TDIM_1 is highlighted in blue colour and green for CG_1. (**b) **Gene annotations to SA fragments across chromosome 1, the pie charts show the percentage of the most representative genes present in each SA fragment, blue and green lines represent segments found in TDIM_1 and CG_1 respectively, the intermedium region between SA fragments is covered by the light red box.

Consistent disomy was observed in most of the clones (Fig. S2). However, divergent AAF distributions of Colombian TcI clones are consistent with the presence of segmental aneuploidy (SA) (diploid, triploid or tretaploid) (Fig. 3). Segmental aneuploidy corresponds to changes in the number of copies of one or various large segments on the extension of disomic chromosomes that do not represent the whole chromosome ploidy. Somy estimations were inferred from AAF distributions in combination with median read-depth variation. The chromosome showing the greatest of degree of segmental aneuploidy (and the longest tracts of consistently raised ploidy) was chromosome 1, this chromosome has been described as central, largely made up of core compartment. The extent of SA on chromosome 1 was perhaps unsurprising considering that this chromosome has the largest areas well-mapped, non-repetitive sequence in which AAF could be evaluated. Tree patterns of SA were found among the clones CG-1, TDIM-1 and V3 in the chromosome 1(Fig. 3a and S2). In the clones TDIM-1 and V3, however, both AAF and read depth suggest segmental trisomy in Ch1 (Fig. 3a). Finally, we found AAF consistent with trisomic SA that was not reflected in the segmental depth increase to CG 1 clone (Fig. 3a). Among other clones, several patterns of segmental aneuploidy among genetically similar clones of the same strain were observed (Fig. S2).

Notably, segmental breakpoint locations were consistent within and among strains on chromosome 1, which led us to examine attributes and annotations of genes and sequences within these regions that correspond with trisomic segments (Fig. 3b). To achieve this, two of the longest segments were selected, i) 1bp- 608394 bp, and ii) 651273 bp to 1409881 bp. We also explored a SA breakpoint in the central section of this region at, 608394 to 1409881 bp (Fig. 3b). We observed a large number of retrotransposon hot spot genes (RHS) in the flanking regions between these segments. Within these segments, RHS were also observed, alongside hypothetical proteins, transialidases, mucins, dispersed gene family proteins, ABC transporters, and ribosomal proteins (Fig. 3b).

We mapped our reads onto small scaffolds reported by Wang *et al.,* 2021 [15] corresponding to alternative allele frequency, and we noted correspondence between zones of SA reported by these authors and our own data (Table S2).

### Differential loss of heterozygosity between clones of *T. cruzi* I suggest frequent mitotic gene conversion events

**Fig. 4.**
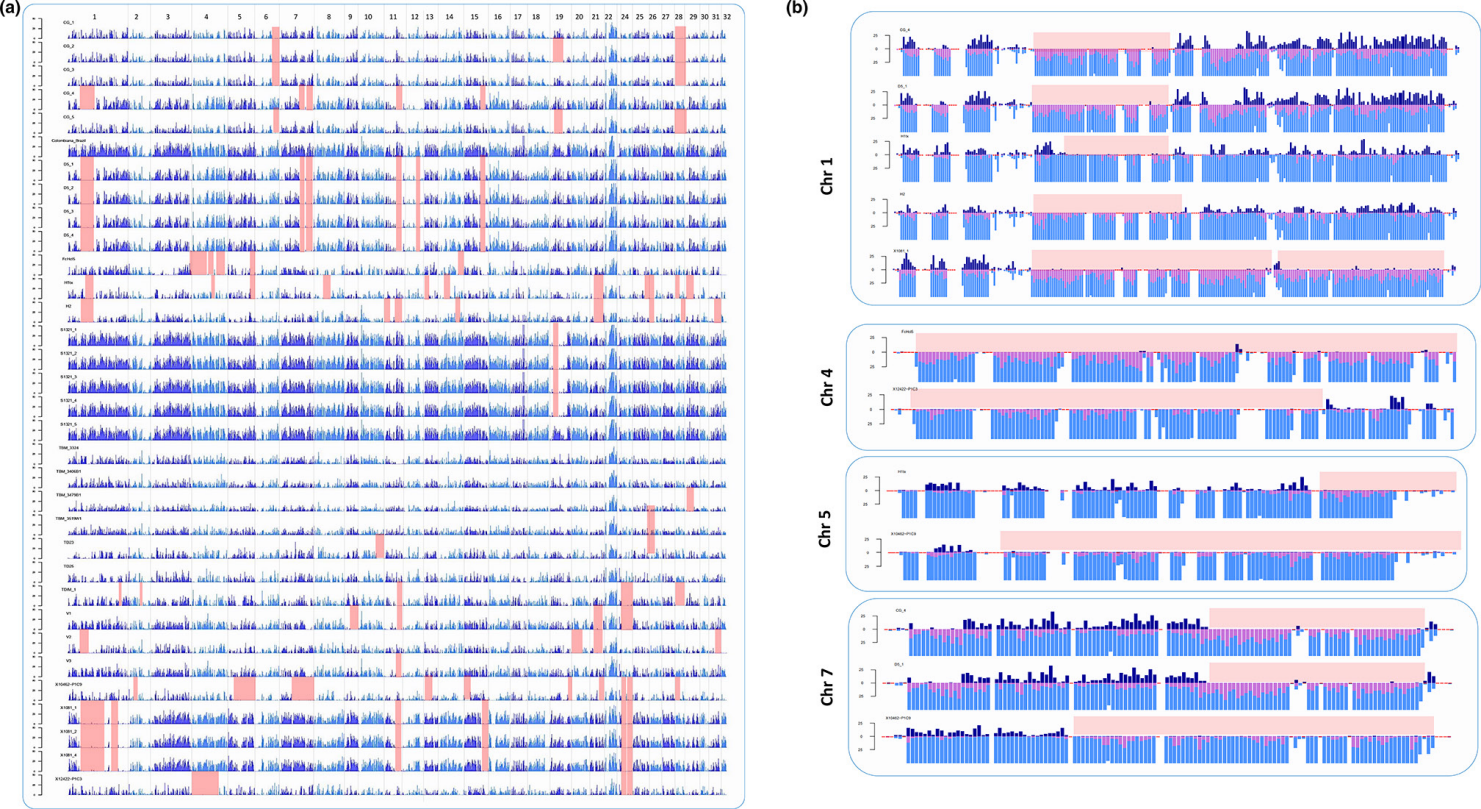
Differential loss of heterozygosity between clones of *T. cruzi* I. (a) Presence of heterozygous SNPs in the first 34 chromosomes, red squares symbolize areas with LOH, x axis represents each chromosome and y axis each clone included in this study** (b) **The bar graph shows the heterozygous SNPs above the y axis (dark blue), homozygous SNPs below the y axis, in two groups, not present in the genome. Reference (pink), present in the reference genome (light blue), red squares symbolize LOH areas.

An analysis of loss of heterozygosity was performed for all the genomes in 10 kb windows, demonstrating the presence of LOH zones along the whole *T. cruzi* genome (Fig. 4a). LOH was not related with previously calculated depth indicating our results were not influenced by coverage because of Illumina performance. LOH were found on disomic and trisomic chromosomes, indicating homozygous copies in the segments described below. We focused our analysis on the most extended segments with LOH, which were located on chromosomes 1, 4, 5 and 7 (Fig. 4b): In chromosome 1, we found LOH in four different segmental patterns in CG-4, Colombiana-brazil, FcHcl5, H1tx, H2, V2, and all the clones from strains D5 and X1081 (Fig. 4b); for chromosome 4, the LOH patterns were found in X12422-P1C3 and FcHc15, where LOH was covering the entire chromosome; the LOH patterns for chromosome 5 were mainly found in H1tx and X10462-P1C9; and for chromosome 7, only one LOH pattern was detected in all the clones of the D5 strain, CG 4 and X10462-P1C9.

The influence of repetitive regions and regions such as RHS in the patterns with LOH was assessed for the chromosomes mentioned above. Segmental LOH was identified on chromosome 1, again flanked by RHS. Our analysis did not show any other relationship between LOH and specific genes. In the case of the chromosome 4, we did not determine the genes that flanked the LOH area because it covers the whole chromosome. Finally, LOH segments in chromosomes 5 and 7 were flanked by genes encoding hypothetical proteins (Fig. S3).

## Discussion

Our analysis of 33 clones indicates a high level of genetic diversity in Colombian TcI clones, with some phylogeographic structuring and the presence of divergent clones within the same host or vector. After mapping to a recently assembled reference genome [15], we were able to demonstrate substantial ploidy instability across the dataset, including several instances of segmental aneuploidy across multiple clones on chromosome 1. We also identified the presence of retrotransposon hotspots, which could have a role in driving genomic re-arrangements. Finally, widespread instances of Loss of Heterozygosity (LOH) were detected and suggest an important role for gene conversion.

The observed results corroborate previous findings associated with human infection clonal genotype TcI_dom_ at the genomic level [6, 8, 48, 49]. A possible ‘bottleneck’ event resulting in a decrease in its genetic variability has been hypothesized for this genotype, and our results show low diversity and the lowest difference in SNPs comparison against the reference genome coherent with the previous hypothesis [5]. This could explain the similarities observed at a phylogenetic level between the strains within different geographical origins [6, 38, 48, 50]. Our results also support previous reports of multiclonality in the CG strain, isolated from a human patient, and TcI sylvatic strains, related with high genetic variability, however, future genomic studies should include more clones that represent TcI_dom_ [17, 30, 38, 51].

Changes in chromosomal somy appear to be well tolerated in trypanosomatids such as *Leishmania, T. congolense* and *T. cruzi*, conversely, *T. brucei* sub-species have not shown aneuploidy, possibly related to different mechanisms of DNA replication and recombination [29, 31, 32, 52]. Ploidy plasticity is believed to be important in responding to environmental stress during the life cycle and precursors of resistance mechanisms against treatment for some *Leishmania* species [23, 24]. Genomic studies on *T. cruzi* have shown aneuploidy changes after passages during cultivation, the same results have been observed in *Leishmania*. Here, we controlled the number of culture passages after the cloning process, however, our results show the presence of aneuploidy patterns for different chromosomes among TcI clones, indicating a possible influence of culture pressure over genome stability that impacts the number of chromosomes copies, as they are in many pathogenic fungi (Fig. 2) [15, 27, 28, 30, 31, 52–54]. Chromosomal mosaicism has been previously discussed including parasexuality, failed or incomplete meiotic processes and meiosis and posterior reduction in the chromosome copies. Given this possibility, it is important to highlight the recent evidence of meiotic sex in some populations of *T. cruzi* [30] although our collection of clones, subdivided in both space and time, were unsuitable for estimating the frequency of genetic exchange (e.g. [5]). It is unknown how *T. cruzi* regulates aneuploidy processes per chromosome, considering the content of repetitive sequences; and therefore, further research is necessary.

Segmental aneuploidy was found on chromosome 1 between TcI clones flanked by RHS and contained families of repetitive gene protein sequences. However, the high number of hypothetical proteins complicates the understanding of the complete genomic organization of these segments (Fig. 3b). RHS has been recently studied in *T. cruzi* and *T. brucei* where sequences have retrotransposon insertion sites in their 5′ coding region and coincide with large hemizygous regions and tandem amplification events [55, 56]. Unequal crossing-over between non-sister homologous chromatids with retrotransposons involved, could affect large segments of the genome and could serve as the explanation for the origins of the trisomic segments in chromosome 1, where one of the copies is deleted resulting in hemizygous, which has been demonstrated in *T. brucei,* where long regions of hemizygous segments affect VSG and other multigene families [57]. RHS have been identified after single strand cleavage sites that are necessary to activate core homologous recombination (HR) factors and initiate the double strand break repair which is essential for the survival of *T. cruzi* during its life cycle [57, 58].

Phenotypic diversity is rapidly driven by LOH and decreases in allelic diversity are associated with the appearance of recessive alleles that may confer selective advantages in response to different kinds of environmental or temporal stressors [59]. LOH has been previously reported in *Leishmania* and *T. brucei gambiense*, related to recombination and asexuality respectively [53, 60, 61]. In yeast, recombination, repair of double strand DNA breaks, and/or chromosome segregation mechanisms are all associated with the appearance of LOH during host infection as well as during *in vitro* stress [58, 62]. Similar processes could drive the LOH profiles observed in TcI clones. Considering the complex biological cycle of *T. cruzi* that involves multiple variations in environmental stressors, LOH could have a very important role and serve as a checkpoint regulator [63, 64]. It is possible that LOH profile variations arose from repair of a double strand DNA break as occurs in *Saccharomyces cerevisiae* [62].

Analysis of the 18 *T*. *cruzi* I clones’ nuclear genomes demonstrated the presence of high genomic plasticity within TcI populations. In some respects, a similar pattern emerges to the observed in *Leishmania* sp. Unlike in *Leishmania* sp, however, the drivers of genome plasticity in *T. cruzi* are far from clear. Does aneuploidy arise in response of environmental stressors for example, or could it be a relic of non-Mendelian or otherwise unorthodox genetic exchange? There is now an urgent need to study genome plasticity in *T. cruzi* under controlled conditions to explore its role in parasite biology, especially with respect to adaptation to new environments, drugs, vectors, hosts and immune pressure.

## Supplementary Data

Supplementary material 1
