# Corrigendum: Genome plasticity driven by aneuploidy and loss of heterozygosity in *Trypanosoma cruzi*

**DOI:** 10.1099/mgen.0.000873

**Published:** 2022-07-20

**Authors:** Lissa Cruz-Saavedra, Philipp Schwabl, Gustavo A. Vallejo, Julio C. Carranza, Marina Muñoz, Luz Helena Patino, Alberto Paniz-Mondolfi, Martin S. Llewellyn, Juan David Ramírez

**Affiliations:** ^1^​ Centro de Investigaciones en Microbiología y Biotecnología-UR (CIMBIUR), Facultad de Ciencias Naturales, Universidad del Rosario, Bogotá, Colombia; ^2^​ Institute of Biodiversity, Animal Health & Comparative Medicine, University of Glasgow, Glasgow G12 8QQ, UK; ^3^​ Laboratorio de Investigación en Parasitología Tropical, Facultad de Ciencias, Universidad del Tolima, Ibagué, Colombia; ^4^​ Molecular Microbiology Laboratory, Department of Pathology, Molecular and Cell-Based Medicine, Icahn School of Medicine at Mount Sinai, New York, NY 10029, USA

## Full-Text

In the published version of this article, the accompanying Supplementary Material did not contain figure legends. The Supplementary Material should have included legends, the correct version of this file can be found associated with this corrigendum.

The author apologizes for any inconvenience caused.

## Supplementary Data

Supplementary material 1

