# Supplementary material for: Corrigendum: Genome plasticity driven by aneuploidy and loss of heterozygosity in Trypanosoma cruzi
Source: Microb Genom. 2022 Jul 20;8(7):mgen000873. doi: 10.1099/mgen.0.000873 (PMC9455697; doi:10.1099/mgen.0.000873)
Supplement: Supplementary material 1 [file mgen-8-873-s001.pdf]

Genome plasticity driven by aneuploidy and loss of heterozygosity in *Trypanosoma cruzi*.

Lissa Cruz-Saavedra[a], Philipp Schwabl[b], Gustavo A. Vallejo[c], Julio C. Carranza[c], Marina Muñoz[a], Luz Helena Patino[a], Alberto Paniz-Mondolfi[d], Martin S. Llewellyn[b], Juan David Ramírez [a,d*].

[a]Centro de Investigaciones en Microbiología y Biotecnología-UR (CIMBIUR), Facultad de Ciencias Naturales, Universidad del Rosario, Bogotá, Colombia.

[b]Institute of Biodiversity, Animal Health & Comparative Medicine, University of Glasgow, Glasgow G12 8QQ, UK.

[c]Laboratorio de Investigación en Parasitología Tropical, Facultad de Ciencias, Universidad del Tolima, Ibagué, Colombia.

[d]Molecular Microbiology Laboratory, Department of Pathology, Molecular and Cell-Based Medicine, Icahn School of Medicine at Mount Sinai, New York, NY 10029, USA

*Correspondence: juand.ramirez@urosario.edu.co

Supporting data – Supplemental material

Fig. S1. Chromosomal Aneuploidy in *T. cruzi* I clones – Dendrogram.

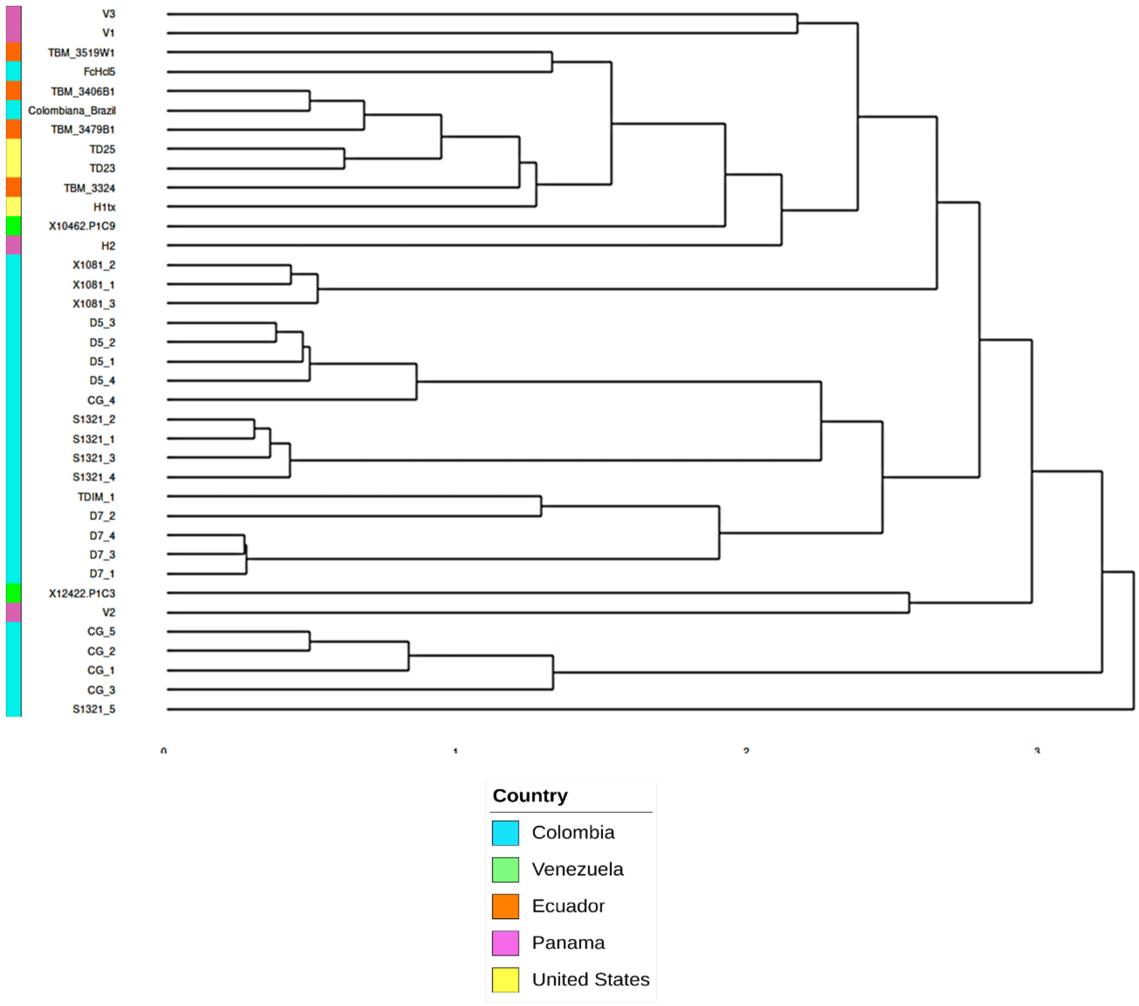

Fig. S2. Allelic frequency and depth per chromosome for all *Trypanosoma cruzi* I included in the study.

PDF file

Access link:

https://figshare.com/s/6957543e5ff02af12837

Fig. S3. Annotation gene of segment with loss of heterozygosity on chromosome 1, 4, 5 and 7.

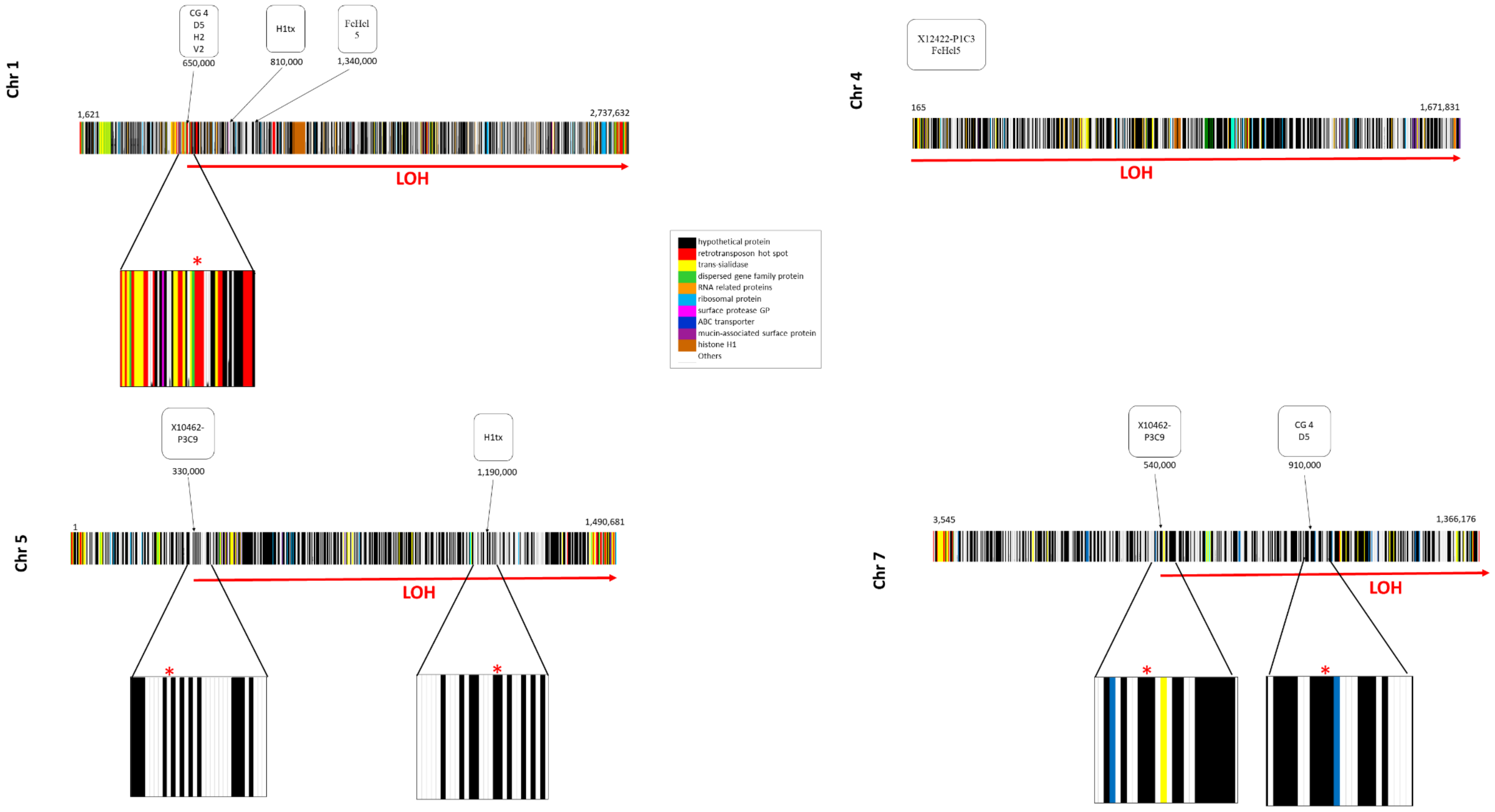

Table. S1. Strain - Clones origin.

Strain-Clone	Origin	Isolate	Study strain
D5	Colombia	*D. marsupialis*	Yes
1321	Colombia	*R. pallescens*	Yes
CG	Colombia	*Human*	Yes
X1081	Colombia	*R. prolixus*	Yes
TDIM	Colombia	*T. dimidiata*	Yes
Colombiana_Brazil	Colombia	*Human*	No
FcHcl5	Colombia	*Human*	No
TBM_3479B1	Ecuador	*R. ecuatoriensis*	No
TBM_3406B1	Ecuador	*R. ecuatoriensis*	No
TBM_3324	Ecuador	*R. ecuatoriensis*	No
TBM_3519W1	Ecuador	*R. ecuatoriensis*	No
H2	Panama	*Human*	No
V1	Panama	*P. geniculatus*	No
V2	Panama	*R. pallescens*	No
V3	Panama	*T. dimidiata*	No
TD23	Texas	*T. dimidiata*	No
TD25	Texas	*T. dimidiata*	No
H1tx	Texas	*T. dimidiata*	No
X10462-P1C9	Venezuela	*Human*	No
X12422-P1C3	Venezuela	*Human*	No

Table. S2. SNPs differences between clones and strains.

	CG_1	CG_2	CG_3	CG_4	CG_5	Colombiana_Brazil	D5_1	D5_2	D5_3	D5_4	FcHcl5	H1tx	H2	S1321_1	S1321_2	S1321_3	S1321_4	S1321_5	TBM_3324	TBM_3406B1	TBM_3479B1	TBM_3519W1	TD23	TD25	TDIM_1	V1	V2	V3	X10462-P1C9	X1081_1	X1081_2	X1081_3	X12422-P1C3
CG_1	0	1198	828	58312	1221	71301	61547	61467	61543	61699	48447	23261	30195	73290	72886	73160	72907	72907	43618	45868	30538	41005	26626	26943	56419	40229	28487	41860	17972	63886	63913	63637	17053
CG_2	1198	0	1142	58200	763	71315	61457	61371	61445	61617	48369	23191	30227	73166	72789	73037	72760	72880	43545	45832	30493	40935	26657	26992	56287	40127	28479	41791	17928	63876	63897	63650	17092
CG_3	828	1142	0	58390	1208	71462	61662	61584	61660	61795	48587	23322	30271	73452	73061	73304	73049	73060	43692	46019	30576	41019	26840	27104	56546	40275	28529	41946	18018	64049	64081	63822	17090
CG_4	58312	58200	58390	0	58138	69153	2396	2373	2406	2389	53547	51705	50757	71242	71008	71109	71068	71031	49591	51803	35083	47218	55655	56672	35619	53455	49856	55046	55661	64378	64319	64259	56234
CG_5	1221	763	1208	58138	0	71234	61322	61250	61325	61488	48268	23108	30200	73054	72673	72915	72653	72769	43522	45752	30435	40889	26567	26889	56210	40135	28423	41771	17890	63773	63811	63543	17040
Colombiana_Brazil	71301	71315	71462	69153	71234	0	71517	71499	71538	71724	66565	65310	63477	4083	4064	4060	3924	3250	58846	61320	41323	55975	69309	70325	67004	65337	62413	67257	69404	72682	72596	72529	69840
D5_1	61547	61457	61662	2396	61322	71517	0	1256	1274	1193	55882	54558	53212	73497	73224	73365	73294	73245	51280	53944	36034	48713	58976	60085	36738	55517	52246	57404	58926	66532	66492	66359	59467
D5_2	61467	61371	61584	2373	61250	71499	1256	0	1278	1235	55783	54485	53127	73424	73173	73309	73247	73219	51241	53853	35982	48643	58866	59923	36658	55486	52161	57360	58837	66520	66509	66343	59384
D5_3	61543	61445	61660	2406	61325	71538	1274	1278	0	1232	55857	54554	53180	73426	73167	73312	73229	73207	51271	53931	36012	48679	59007	60039	36632	55526	52248	57414	58897	66534	66542	66398	59482
D5_4	61699	61617	61795	2389	61488	71724	1193	1235	1232	0	55938	54697	53321	73579	73332	73498	73391	73352	51358	54072	36097	48761	59151	60261	36782	55654	52386	57537	59089	66638	66636	66489	59658
FcHcl5	48447	48369	48587	53547	48268	66565	55882	55783	55857	55938	0	43557	42518	67745	67533	67661	67454	67472	43636	46058	30903	41272	47272	48127	50975	45283	42228	47024	46385	60535	60458	60359	47070
H1tx	23261	23191	23322	51705	23108	65310	54558	54485	54554	54697	43557	0	28724	65541	65241	65434	65277	65299	40311	42154	28698	38188	22051	21609	50059	37501	27317	38875	21052	57088	57092	56940	21426
H2	30195	30227	30271	50757	30200	63477	53212	53127	53180	53321	42518	28724	0	63677	63448	63633	63411	63452	39441	41037	28313	37493	31484	31626	48944	35424	20088	36397	29752	55792	55769	55649	29935
S1321_1	73290	73166	73452	71242	73054	4083	73497	73424	73426	73579	67745	65541	63677	0	1472	1514	1527	2362	58883	61555	41259	55987	69814	70943	68747	65675	62408	67758	70748	74473	74405	74367	71159
S1321_2	72886	72789	73061	71008	72673	4064	73224	73173	73167	73332	67533	65241	63448	1472	0	1510	1503	2321	58751	61301	41186	55861	69444	70515	68546	65420	62248	67487	70362	74194	74123	74085	70815
S1321_3	73160	73037	73304	71109	72915	4060	73365	73309	73312	73498	67661	65434	63633	1514	1510	0	1499	2386	58841	61475	41237	55897	69672	70758	68694	65557	62402	67626	70586	74334	74271	74221	71037
S1321_4	72907	72760	73049	71068	72653	3924	73294	73247	73229	73391	67454	65277	63411	1527	1503	1499	0	2343	58696	61294	41154	55823	69448	70565	68572	65414	62220	67483	70403	74214	74137	74106	70841
S1321_5	72907	72880	73060	71031	72769	3250	73245	73219	73207	73352	67472	65299	63452	2362	2321	2386	2343	0	58789	61373	41211	55887	69496	70537	68513	65460	62244	67507	70394	74199	74141	74099	70786
TBM_3324	43618	43545	43692	49591	43522	58846	51280	51241	51271	51358	43636	40311	39441	58883	58751	58841	58696	58789	0	24350	17642	22275	42581	43042	46982	41144	38971	41954	41687	54202	54174	54162	42303
TBM_3406B1	45868	45832	46019	51803	45752	61320	53944	53853	53931	54072	46058	42154	41037	61555	61301	61475	61294	61373	24350	0	17295	23656	44668	45473	49380	42880	40233	43876	43995	57067	56993	56902	44606
TBM_3479B1	30538	30493	30576	35083	30435	41323	36034	35982	36012	36097	30903	28698	28313	41259	41186	41237	41154	41211	17642	17295	0	17186	29872	30104	33323	29550	27901	29957	29201	38059	38006	37960	29711
TBM_3519W1	41005	40935	41019	47218	40889	55975	48713	48643	48679	48761	41272	38188	37493	55987	55861	55897	55823	55887	22275	23656	17186	0	40219	40598	44779	39193	37064	39846	39267	51429	51344	51326	39746
TD23	26626	26657	26840	55655	26567	69309	58976	58866	59007	59151	47272	22051	31484	69814	69444	69672	69448	69496	42581	44668	29872	40219	0	18376	53999	39744	29379	41312	26053	61563	61554	61365	25887
TD25	26943	26992	27104	56672	26889	70325	60085	59923	60039	60261	48127	21609	31626	70943	70515	70758	70565	70537	43042	45473	30104	40598	18376	0	54811	40295	29569	41887	26297	62880	62835	62611	26203
TDIM_1	56419	56287	56546	35619	56210	67004	36738	36658	36632	36782	50975	50059	48944	68747	68546	68694	68572	68513	46982	49380	33323	44779	53999	54811	0	51140	47874	52898	53785	61455	61393	61331	54526
V1	40229	40127	40275	53455	40135	65337	55517	55486	55526	55654	45283	37501	35424	65675	65420	65557	65414	65460	41144	42880	29550	39193	39744	40295	51140	0	34851	2705	38877	58314	58265	58139	39424
V2	28487	28479	28529	49856	28423	62413	52246	52161	52248	52386	42228	27317	20088	62408	62248	62402	62220	62244	38971	40233	27901	37064	29379	29569	47874	34851	0	35844	28284	54792	54753	54597	28300
V3	41860	41791	41946	55046	41771	67257	57404	57360	57414	57537	47024	38875	36397	67758	67487	67626	67483	67507	41954	43876	29957	39846	41312	41887	52898	2705	35844	0	40638	60211	60172	60031	41156
X10462-P1C9	17972	17928	18018	55661	17890	69404	58926	58837	58897	59089	46385	21052	29752	70748	70362	70586	70403	70394	41687	43995	29201	39267	26053	26297	53785	38877	28284	40638	0	61229	61216	60987	5016
X1081_1	63886	63876	64049	64378	63773	72682	66532	66520	66534	66638	60535	57088	55792	74473	74194	74334	74214	74199	54202	57067	38059	51429	61563	62880	61455	58314	54792	60211	61229	0	1139	1202	61733
X1081_2	63913	63897	64081	64319	63811	72596	66492	66509	66542	66636	60458	57092	55769	74405	74123	74271	74137	74141	54174	56993	38006	51344	61554	62835	61393	58265	54753	60172	61216	1139	0	1181	61745
X1081_3	63637	63650	63822	64259	63543	72529	66359	66343	66398	66489	60359	56940	55649	74367	74085	74221	74106	74099	54162	56902	37960	51326	61365	62611	61331	58139	54597	60031	60987	1202	1181	0	61490
X12422-P1C3	17053	17092	17090	56234	17040	69840	59467	59384	59482	59658	47070	21426	29935	71159	70815	71037	70841	70786	42303	44606	29711	39746	25887	26203	54526	39424	28300	41156	5016	61733	61745	61490	0

Table. S3. Number of reads present in segmental allelic frequency patterns on contigs described by Wang *et al*., 2021 that had synteny with chromosome 1.

TcBrA4_Contig97

Strain	Contig number	Chromosome synteny	Start	End	Number of reads
CG_1	Contig97	Chr1	2076453	2097662	1828
CG_2	Contig97	Chr1	2076453	2097662	2067
CG_3	Contig97	Chr1	2076453	2097662	2218
CG_4	Contig97	Chr1	2076453	2097662	1659
CG_5	Contig97	Chr1	2076453	2097662	2043
Colombiana_Brazil	Contig97	Chr1	2076453	2097662	2749
D5_1	Contig97	Chr1	2076453	2097662	3047
D5_2	Contig97	Chr1	2076453	2097662	3294
D5_3	Contig97	Chr1	2076453	2097662	3209
D5_4	Contig97	Chr1	2076453	2097662	3038
FcHcl5	Contig97	Chr1	2076453	2097662	3518
H1tx	Contig97	Chr1	2076453	2097662	1249
H2	Contig97	Chr1	2076453	2097662	966
S1321_1	Contig97	Chr1	2076453	2097662	3275
S1321_2	Contig97	Chr1	2076453	2097662	2921
S1321_3	Contig97	Chr1	2076453	2097662	3145
S1321_4	Contig97	Chr1	2076453	2097662	2850
S1321_5	Contig97	Chr1	2076453	2097662	3064
TBM_3324	Contig97	Chr1	2076453	2097662	982
TBM_3406B1	Contig97	Chr1	2076453	2097662	1517
TBM_3479B1	Contig97	Chr1	2076453	2097662	404
TBM_3519W1	Contig97	Chr1	2076453	2097662	685
TD23	Contig97	Chr1	2076453	2097662	1960
TD25	Contig97	Chr1	2076453	2097662	1631
TDIM_1	Contig97	Chr1	2076453	2097662	3588
TDIM_2	Contig97	Chr1	2076453	2097662	3767
V1	Contig97	Chr1	2076453	2097662	1170
V2	Contig97	Chr1	2076453	2097662	1036
V3	Contig97	Chr1	2076453	2097662	1685
X10462-P1C9	Contig97	Chr1	2076453	2097662	2314
X1081_1	Contig97	Chr1	2076453	2097662	3180
X1081_2	Contig97	Chr1	2076453	2097662	3133
X1081_3	Contig97	Chr1	2076453	2097662	2990
X12422-P1C3	Contig97	Chr1	2076453	2097662	2370

TcBrA4_Contig345

Strain	Contig number	Chromosome synteny	Start	End	Numer of reads
CG_1	TcBrA4_Contig345	Chr1	66388	68894	300
CG_2	TcBrA4_Contig345	Chr1	66388	68894	282
CG_3	TcBrA4_Contig345	Chr1	66388	68894	320
CG_4	TcBrA4_Contig345	Chr1	66388	68894	349
CG_5	TcBrA4_Contig345	Chr1	66388	68894	302
Colombiana_Brazil	TcBrA4_Contig345	Chr1	66388	68894	608
D5_1	TcBrA4_Contig345	Chr1	66388	68894	317
D5_2	TcBrA4_Contig345	Chr1	66388	68894	356
D5_3	TcBrA4_Contig345	Chr1	66388	68894	340
D5_4	TcBrA4_Contig345	Chr1	66388	68894	387
FcHcl5	TcBrA4_Contig345	Chr1	66388	68894	926
H1tx	TcBrA4_Contig345	Chr1	66388	68894	234
H2	TcBrA4_Contig345	Chr1	66388	68894	494
S1321_1	TcBrA4_Contig345	Chr1	66388	68894	429
S1321_2	TcBrA4_Contig345	Chr1	66388	68894	321
S1321_3	TcBrA4_Contig345	Chr1	66388	68894	373
S1321_4	TcBrA4_Contig345	Chr1	66388	68894	370
S1321_5	TcBrA4_Contig345	Chr1	66388	68894	465
TBM_3324	TcBrA4_Contig345	Chr1	66388	68894	280
TBM_3406B1	TcBrA4_Contig345	Chr1	66388	68894	358
TBM_3479B1	TcBrA4_Contig345	Chr1	66388	68894	157
TBM_3519W1	TcBrA4_Contig345	Chr1	66388	68894	198
TD23	TcBrA4_Contig345	Chr1	66388	68894	441
TD25	TcBrA4_Contig345	Chr1	66388	68894	615
TDIM_1	TcBrA4_Contig345	Chr1	66388	68894	306
TDIM_2	TcBrA4_Contig345	Chr1	66388	68894	342
V1	TcBrA4_Contig345	Chr1	66388	68894	362
V2	TcBrA4_Contig345	Chr1	66388	68894	250
V3	TcBrA4_Contig345	Chr1	66388	68894	569
X10462-P1C9	TcBrA4_Contig345	Chr1	66388	68894	577
X1081_1	TcBrA4_Contig345	Chr1	66388	68894	341
X1081_2	TcBrA4_Contig345	Chr1	66388	68894	358
X1081_3	TcBrA4_Contig345	Chr1	66388	68894	310
X12422-P1C3	TcBrA4_Contig345	Chr1	66388	68894	612

TcBrA4_Contig347

Strain	Contig number	Chromosome synteny	Start	End	Numer of reads
CG_1	TcBrA4_Contig347	Chr1	1973980	1991624	794
CG_2	TcBrA4_Contig347	Chr1	1973980	1991624	829
CG_3	TcBrA4_Contig347	Chr1	1973980	1991624	953
CG_4	TcBrA4_Contig347	Chr1	1973980	1991624	160
CG_5	TcBrA4_Contig347	Chr1	1973980	1991624	752
Colombiana_Brazil	TcBrA4_Contig347	Chr1	1973980	1991624	1404
D5_1	TcBrA4_Contig347	Chr1	1973980	1991624	556
D5_2	TcBrA4_Contig347	Chr1	1973980	1991624	476
D5_3	TcBrA4_Contig347	Chr1	1973980	1991624	501
D5_4	TcBrA4_Contig347	Chr1	1973980	1991624	543
FcHcl5	TcBrA4_Contig347	Chr1	1973980	1991624	1325
H1tx	TcBrA4_Contig347	Chr1	1973980	1991624	561
H2	TcBrA4_Contig347	Chr1	1973980	1991624	496
S1321_1	TcBrA4_Contig347	Chr1	1973980	1991624	1121
S1321_2	TcBrA4_Contig347	Chr1	1973980	1991624	996
S1321_3	TcBrA4_Contig347	Chr1	1973980	1991624	1145
S1321_4	TcBrA4_Contig347	Chr1	1973980	1991624	899
S1321_5	TcBrA4_Contig347	Chr1	1973980	1991624	1367
TBM_3324	TcBrA4_Contig347	Chr1	1973980	1991624	422
TBM_3406B1	TcBrA4_Contig347	Chr1	1973980	1991624	633
TBM_3479B1	TcBrA4_Contig347	Chr1	1973980	1991624	244
TBM_3519W1	TcBrA4_Contig347	Chr1	1973980	1991624	376
TD23	TcBrA4_Contig347	Chr1	1973980	1991624	849
TD25	TcBrA4_Contig347	Chr1	1973980	1991624	1438
TDIM_1	TcBrA4_Contig347	Chr1	1973980	1991624	578
TDIM_2	TcBrA4_Contig347	Chr1	1973980	1991624	692
V1	TcBrA4_Contig347	Chr1	1973980	1991624	673
V2	TcBrA4_Contig347	Chr1	1973980	1991624	327
V3	TcBrA4_Contig347	Chr1	1973980	1991624	926
X10462-P1C9	TcBrA4_Contig347	Chr1	1973980	1991624	1420
X1081_1	TcBrA4_Contig347	Chr1	1973980	1991624	968
X1081_2	TcBrA4_Contig347	Chr1	1973980	1991624	1009
X1081_3	TcBrA4_Contig347	Chr1	1973980	1991624	1013
X12422-P1C3	TcBrA4_Contig347	Chr1	1973980	1991624	1387
